# *Bacillus cereus* AR156 triggers induced systemic resistance against *Pseudomonas syringae* pv. *tomato* DC3000 by suppressing miR472 and activating CNLs‐mediated basal immunity in *Arabidopsis*

**DOI:** 10.1111/mpp.12935

**Published:** 2020-03-30

**Authors:** Chunhao Jiang, Zhihang Fan, Zijie Li, Dongdong Niu, Yan Li, Mingzi Zheng, Qi Wang, Hailing Jin, Jianhua Guo

**Affiliations:** ^1^ Department of Plant Pathology College of Plant Protection Nanjing Agricultural University Nanjing China; ^2^ Key Laboratory of Monitoring and Management of Crop Diseases and Pest Insects Ministry of Agriculture Nanjing China; ^3^ Engineering Center of Bioresource Pesticide in Jiangsu Province Nanjing China; ^4^ Department of Plant Pathology College of Plant Protection China Agricultural University Beijing China; ^5^ Department of Plant Pathology and Microbiology University of California Riverside CA USA

**Keywords:** *Arabidopsis*, *Bacillus cereus*, induced systemic resistance, microRNA, plant innate immunity, *Pseudomonas syringae* pv. *tomato*

## Abstract

Small RNAs play an important role in plant innate immunity. However, their regulatory function in induced systemic resistance (ISR) triggered by plant growth‐promoting rhizobacteria remains unclear. Here, using *Arabidopsis* as a model system, one plant endogenous small RNA, miR472, was identified as an important regulator involved in the process of *Bacillus cereus* AR156 ISR against *Pseudomonas syringae* pv. *tomato* (Pst) DC3000. The results revealed that miR472 was down‐regulated with *B. cereus* AR156 treatment by comparing small RNA profiles and northern blot analysis of *Arabidopsis* with or without *B. cereus* AR156 treatment. Plants overexpressing miR472 showed higher susceptibility to Pst DC3000; by contrast, plant lines with miR472 knocked down/out showed the opposite. The transcriptome sequencing revealed thousands of differentially expressed genes in the transgenic plants. Target prediction showed that miR472 targets lots of coiled coil nucleotide‐binding site (NBS) and leucine‐rich repeat (LRR) type resistance genes and the expression of these targets was negatively correlated with the expression of miR472. In addition, transgenic plants with knocked‐out target genes exhibited decreased resistance to Pst DC3000 invasion. Quantitative reverse transcription PCR results indicated that target genes of miR472 were expressed during the process of *B. cereus* AR156‐triggered ISR. Taken together, our results demonstrate that the miR472‐mediated silencing pathway is an important regulatory checkpoint occurring via post‐transcriptional control of NBS‐LRR genes during *B. cereus* AR156‐triggered ISR in *Arabidopsis*.

## Graphical abstract

The plant miR472‐mediated silencing pathway is an important regulatory checkpoint occurring via post‐transcriptional control of nucleotide‐binding site and leucine‐rich repeat genes during *Bacillus cereus* AR156‐triggered induced systemic resistance in *Arabidopsis*.

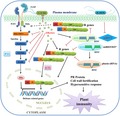

**Abbreviations**
CNL: CC‐NBS‐LRR
DEG: differentially expressed gene
ET: ethylene
ETI: effector‐triggered immunity
GO: gene ontology
ISR: induced systemic resistance
JA: jasmonic acid
KEGG: Kyoto Encyclopaedia of Genes and Genomes enrichments
MAMPS/PAMPs: microbial‐ or pathogen‐associated molecular patterns
NLR: nucleotide‐binding leucine‐rich repeat receptor class
PGPR: plant growth‐promoting rhizobacteria
PRR: pattern recognition receptor
PTI: PAMP‐triggered immunity
R gene: plant disease resistance gene
RLP/RLK: receptor‐like protein/kinase class
RPKM: reads per kilobase of exon per million fragments
SA: salicylic acid
SAR: systemic acquired resistance
TNL: TIR‐NBS‐LRR

## 1 INTRODUCTION

To survive in a complex and hostile soil environment, plants have evolved multiple types of inducible immune responses to attacks by pathogens (Niu *et al.*, 2016). The plant immune system has two major branches. The first, called PAMP‐triggered immunity (PTI), uses transmembrane pattern recognition receptors (PRRs) or surface receptors, which encode transmembrane receptor‐like kinases. These kinases recognize conserved microbial‐ or pathogen‐associated molecular patterns (MAMPS or PAMPs) such as flagellin, glycoprotein, and chitin. The perception of PAMPs by PRRs triggers a cascade that includes activation of mitogen‐activated protein kinase (MAPK), oxidative burst and callose deposition, induction of defence‐related genes, and accumulation of antimicrobial compounds (Jones and Dangl, 2006; Schwessinger and Zipfel, 2008; Niu *et al.*, 2016). Some pathogens that have successfully infected the host and caused disease can suppress PTI by secreting effectors. In turn, plants develop a second type of immune response, called effector‐triggered immunity (ETI). The ETI acts largely inside the plant cell via polymorphic nucleotide‐binding domain and leucine‐rich repeat (NB‐LRR) proteins, which are encoded by most plant disease resistance (R) genes (Dangl and Jones, 2001; Speth *et al.*, 2007; Katiyar‐Agarwal and Jin, 2010; Kong *et al.*, 2017). NB‐LRR‐mediated disease resistance is effective against obligate biotrophic and hemibiotrophic pathogens, which can only grow in living host tissue, but not against necrotrophic pathogens, which kill host tissue during colonization (Glazebrook, 2005; Jones and Dangl, 2006).

Depending on their protein structures, plant R proteins are divided into two major classes: receptor‐like protein/kinases (RLP/RLK) and nucleotide‐binding leucine‐rich repeat receptors (NLR) (Baker *et al.*, 1997). RLP/RLK proteins are transmembrane immune receptors containing an N‐terminal LRR and a C‐terminal transmembrane domain with cytoplasmic kinase domain RLKs (Zhou and Yang, 2016). NLR class proteins contain an N‐terminal NB domain and C‐ terminal LRR domain. According to their N‐terminal structure, NLR proteins are divided into two subclasses: TIR‐NB‐LRRs (TNLs), which contain a toll/interleukin‐1 receptor (TIR) domain, and CC‐NB‐LRRs (CNLs), which contain a coiled‐coil domain (Austin *et al.*, 2002). TNLs and CNLs are both intracellular immune receptors and require common signalling components. However, TNLs and CNLs possess differential genetic requirements when mediating ETI. For example, TNLs signal downstream events via EDS1/PAD4/ SAG101, while CNLs signal via nuclear Dbf2‐related kinase‐1 (Cui *et al.*, 2015).

Small RNAs are usually 20–30 nucleotide (nt)‐long noncoding molecules, known as important regulators in plant defence against pathogens (Huang *et al.*, 2016; Niu *et al.*, 2016). In plants, small RNAs are divided into two major classes: small interfering RNAs (siRNA) and microRNAs (miRNA). siRNAs are generated from complementary long double‐stranded RNAs (dsRNAs) and examples consist of hc‐siRNAs, ta‐siRNAs, nat‐siRNAs, lsiRNAs, and so on (Katiyar‐Agarwal and Jin, 2010; Huang *et al.*, 2016). miRNAs are usually 21–24 nt and are derived from RNAs with imperfectly base‐paired hairpin structures (Chen, 2009). To regulate gene expression, both miRNA and siRNA use post‐transcriptional gene silencing (PTGS) via an RNA‐induced silencing complex (RISC), which directly cleaves messenger RNAs (mRNAs) or inhibits their translation. siRNA and certain miRNAs can also regulate gene expression using transcriptional gene silencing (TGS) via DNA methylation, and histone or chromatin modification (Vaucheret, 2006; Wu *et al.*, 2010; Cui and Cao, 2014). Numerous miRNA and siRNA are involved in plant defence against pathogens. For example, miR393, which is inducible by flg22 (a type of PAMP), was the first miRNA shown to be involved in plant innate immunity by suppressing the expression of auxin receptors (Navarro *et al.*, 2006). nat‐siRNAATGB2, the first identified endogenous siRNA, can be specifically induced by *Pseudomonas syringae* pv. *tomato* (Pst) AvrRpt2 and positively regulates RPS2‐mediated ETI responses in *Arabidopsis thaliana* (Katiyar‐Agarwal *et al.*, 2006).

Recently, miRNAs have been shown to negatively regulate the expression of plant R genes (Zhai *et al.*, 2011; Li *et al.*, 2012; Shivaprasad *et al.*, 2012). Genome‐wide analysis indicated that miRNAs can directly target numerous plant R genes. For instance, 171 out of 235 TNLs and 178 out of 290 CNLs are regulated by miRNAs in *Glycine max* (Zhao *et al.*, 2015). Certain R genes with NBS‐LRR motifs could be targeted by miR482 and miR2118. In addition, further research indicated that some of these R genes with NBS‐LRR motifs were cleaved then subsequently generated series of secondary siRNAs in an RDR6‐dependent manner when they were targeted by these 22‐nt miRNAs. The newly generated secondary siRNAs can target mRNAs of several defence‐related proteins in tomato (Li *et al.*, 2012). For example, in *Arabidopsis*, miR472 can modulate both PTI and ETI via post‐transcriptional control of CNL genes (Boccara *et al.*, 2014). In tomato, miR398, miR482, and miR5300 can be significantly induced in resistant tomato cultivars on *Fusarium oxysporum* challenge. Ouyang and associates found that miR482/miR5300‐mediated regulation of R genes plays an important role in tomato resistance to pathogenic fungi (Ouyang *et al.*, 2014).

In plants, local activation of PTI/ETI often triggers resistance in tissues at a site distal from the infection, which enhances resistance against a broad spectrum of pathogens (Pieterse *et al.*, 2014; Niu *et al.*, 2016). Examples of such induced resistance are systemic acquired resistance (SAR) and induced systemic resistance (ISR) (Jiang *et al.*, 2016). SAR is usually triggered by exposure to virulent microbes, and is often associated with increased levels of the hormone salicylic acid (SA) and simultaneous activation of pathogenesis‐related (PR) genes such as *PR1*, *PR2*, and *PR5* (Conrath *et al.*, 2002; Niu *et al.*, 2011, 2016; Pieterse *et al.*, 2014). ISR is activated by nonpathogenic rhizobacteria, such as *Pseudomonas* and *Bacillus*; this mechanism is best characterized by plant growth‐promoting rhizobacteria (PGPR) (Choudhary *et al.*, 2007; Van der Ent *et al.*, 2009; Niu *et al.*, 2011; Jiang *et al.*, 2016). Distinguished from SAR, ISR normally depends on the jasmonic acid (JA)/ethylene (ET) signalling pathways. ISR is also accompanied by high expression of *PR* genes and plant defensin 1.2 (*PDF1.2*) (Van Oosten *et al.*, 2008; Niu *et al.*, 2011; Jiang *et al.*, 2016). ISR, which has been demonstrated in numerous plant species such as *Arabidopsis*, tomato, and cucumber, can protect the host against a broad spectrum of pathogens (Van der Ent *et al.*, 2009; Niu *et al.*, 2011; Jiang *et al.*, 2016). For example, the PGPR *Bacillus cereus* AR156 (hereafter AR156) triggers ISR by simultaneously activating SA and JA/ET signalling pathways in a manner dependent on the expression of *NPR1* (Niu *et al.*, 2011). Although ISR triggered by rhizobacteria has been shown in many plant species, few studies have examined the regulatory mechanisms. Jiang *et al.* identified two transcriptional factors (TFs), WRKY11 and WRKY70, that were identified as the key players in AR156‐triggered ISR to Pst DC3000 (Jiang *et al.*, 2016). In addition to TFs, small RNAs are also involved in regulating systemic immune responses induced by rhizobacteria in plants. Using deep sequencing to analyse small RNA species in plants, Niu *et al.* demonstrated that miR825 and miR825* participate in modulation of AR156‐triggered ISR by negatively regulating numerous R genes; however, whether other small RNAs participate in AR156‐triggered ISR in plants remains undetermined.

In this study, we aimed to identify and characterize small RNAs that regulate systemic immune responses induced by AR156 in plants, therefore we analysed the previously obtained small RNA deep sequencing data. We found that expression of the small RNA miR472 in *Arabidopsis* was significantly down‐regulated by treatment with AR156. Furthermore, the expression of miR472 was significantly suppressed by AR156 upon infection with Pst DC3000. Pathogen assay results indicated that the transgenic plants overexpressing miR472 showed more susceptibility to Pst DC3000. In contrast, plants with knockdown and knockout of miR472 showed more resistance. In addition, compared with the wild type, the phenotypes of ISR triggered by AR156 shown on the above transgenic plants were also significantly affected and the ability of AR156 to trigger ISR was decreased significantly. The results of transcriptome sequencing also revealed hundreds of differentially expressed genes (DEGs) in the transgenic plants overexpressing miR472, and in plants with miR472 knockdown and knockout. We further identified five R genes, probably targeted by miR472, that were involved in the process of AR156‐triggered ISR. Taken together, our study shows that the small RNA miR472 participates in modulating AR156‐triggered ISR to Pst D3000 by negatively regulating several resistance‐related genes in plants.

## 2 RESULTS

### 2.1 Expression of miR472 is differentially regulated in *B. cereus *AR156‐triggered ISR in *Arabidopsis*

**FIGURE 1:**
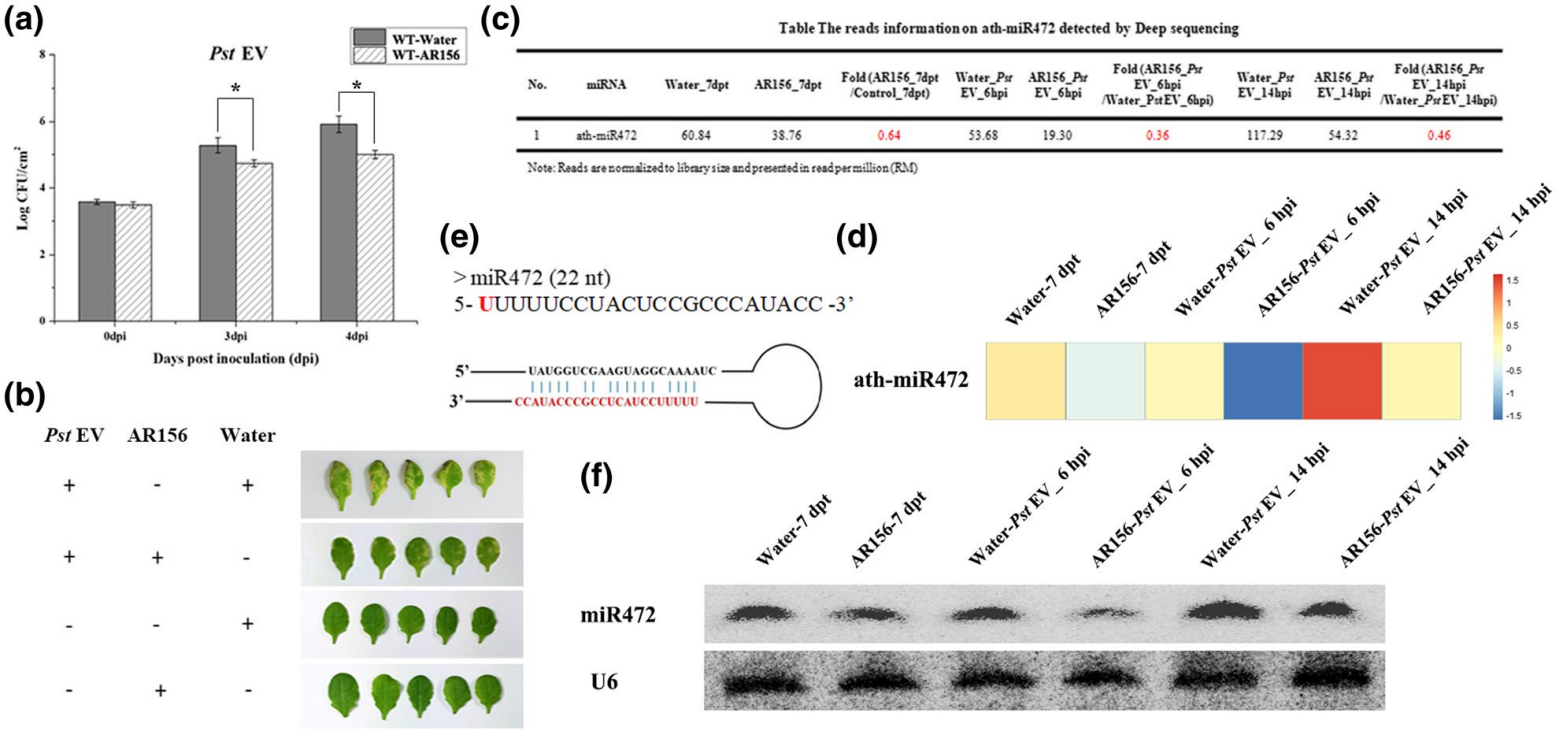
Plant endogenous small RNA miR472 is involved in regulation of *Bacillus cereus* AR156‐triggered induced systemic resistance (ISR) in *Arabidopsis*. (a) Induction of systemic resistance to *Pseudomonas syringae* pv. *tomato* (Pst) DC3000 in *Arabidopsis thaliana* ecotype Col‐0 wild‐type (WT) by AR156. The plants were pretreated with AR156 at 5 × 10^7^ cfu/ml for 5 days and 0.85% NaCl. Subsequently, leaves were spray inoculated with Pst DC3000 (OD_600_ = 0.1). The concentration of Pst DC3000 in leaves and the effect of ISR were measured by the agar plate dilution method at 3 and 4 days post‐inoculation (dpi), respectively. (b) The phenotypic effects of AR156 ISR to Pst DC3000 in *Arabidopsis*. (c) The deep‐sequencing result of miR472 of AR156‐triggered ISR at different time points. The data represent reads per kilobase of exon per million fragments (RPKM) values of miR472 in different samples. (d) Heatmap showing the expression of miR472 in different samples. (e) Predicted secondary stem loop structure for miR472 with mature miR472 sequences indicated. (f) Expression of miR472 in different samples was detected by northern blotting. RNA blots were hybridized with an miR472 probe and U6 was used as a loading control

*B. cereus* AR156 could trigger ISR against Pst DC3000 (Niu *et al.*, 2011); this finding agrees with our results, shown in Figure 1a,b. Transcription factors WRKY70 and WRKY11, and plant small RNAs miR825/825* serve as important regulators during this process (Jiang *et al.*, 2016; Niu *et al.*, 2016). To identify and characterize whether other small RNAs regulate AR156‐triggered ISR, we analysed the small RNA deep sequencing data obtained previously (Niu *et al.*, 2016). We identified one small RNA, miR472, whose expression was significantly down‐regulated by combined treatment with AR156 and Pst DC3000, but only in *Arabidopsis* plants pretreated with AR156 (Figure 1c–e). The expression of miR472, assessed at 6 and 14 hr after challenge with Pst DC3000, was repressed at least 1.5‐fold in plants pretreated with AR156 compared with that pretreated with vehicle control (Figure 1c,d). Northern blotting also indicated that expression of miR472 was significantly down‐regulated in plants administered AR156/Pst DC3000 compared with that in plants administered control/Pst DC3000 (Figure 1f). These findings suggest that miR472 may play an important role in AR156‐triggered ISR.

### 2.2 Transgenic *Arabidopsis* plants with overexpression or knockdown/out of miR472 show an altered defence response

**FIGURE 2:**
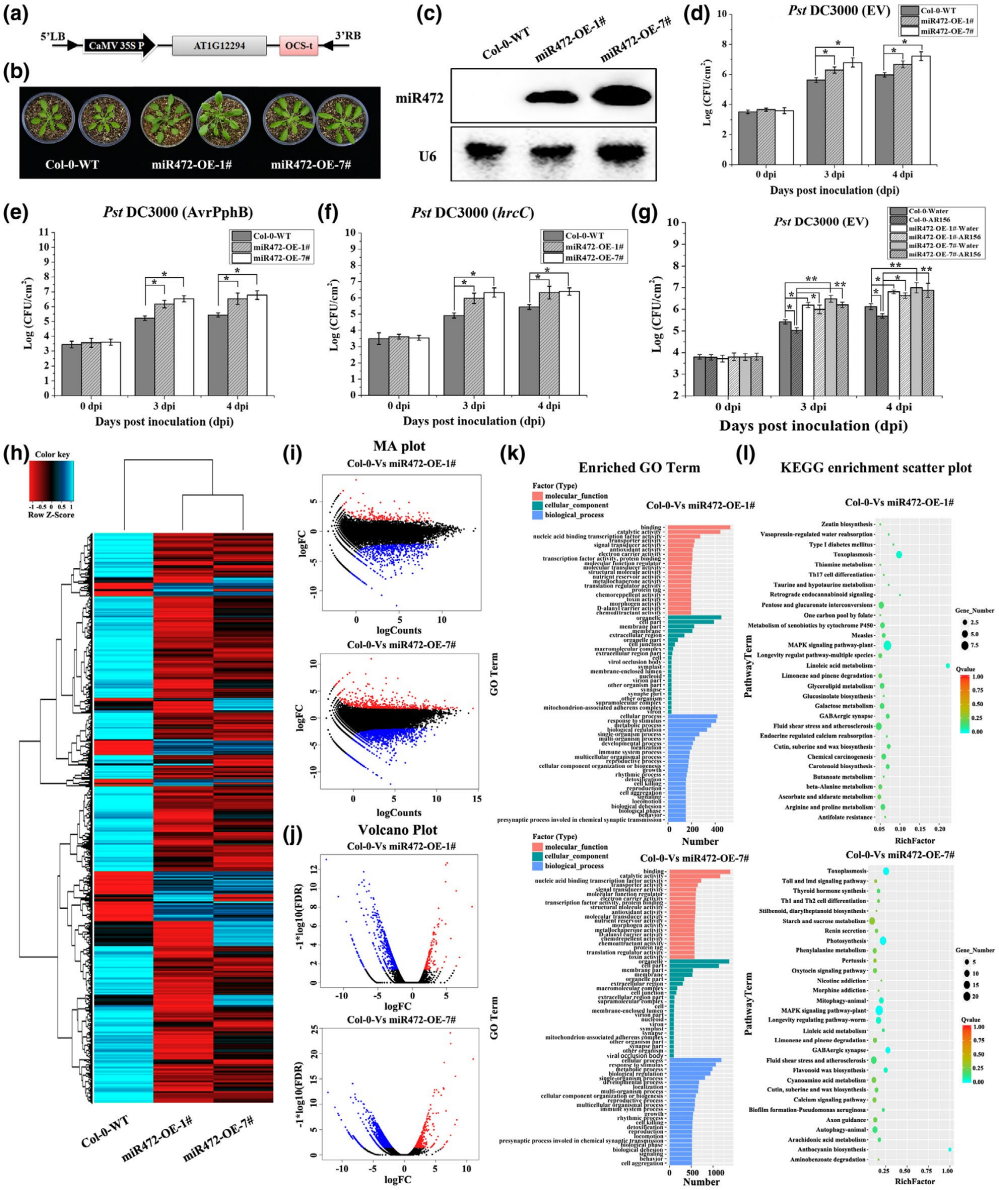
The miR472 overexpression plants are more susceptible to virulent *Pseudomonas syringae* pv. *tomato* (Pst) DC3000. The pathogen assay and induced systemic resistance (ISR) triggered by *Bacillus cereus* AR156 in *Arabidopsis thaliana* ecotype Col‐0 wild‐type (WT) and miR472 overexpression lines (miR472‐OE‐1# and miR472‐OE‐7#) were done as described in the materials and methods section. (a) Schematic diagram of miR472 overexpression constructs used to generate the transgenic plants. (b) Phenotype of miR472 overexpression transgenic plant (line 7#) was compared to ecotype Col‐0 WT, and the images show the 4‐week‐old seedlings. (c). Expression of miR472 in the overexpression lines (miR472‐OE‐1# and miR472‐OE‐7#) by northern blotting with an miR472 probe; U6 was used as a loading control. (d)–(f) Bacterial growth in 5‐week‐old plants from Col‐0 WT, miR472‐OE‐1#, and miR472‐OE‐7# infiltrated with Pst DC3000 (empty vector, EV) (OD_600_ = 0.0001), Pst DC3000 (*AvrPphB*) (OD_600_ = 0.0005), and Pst DC3000 (*hrcC*) (OD_600_ = 0.0001), respectively. (g) Bacterial growth assay of Pst DC3000 (EV) in Col‐0 WT, miR472‐OE‐1#, and miR472‐OE‐7# pretreated with AR156 or 0.85% NaCl. For (d)–(g), the data are means and *SD* (*n* = 24). The LSD test was performed to determine the significant differences between miR472 OE lines (miR472‐OE‐1# and miR472‐OE‐7#) and Col‐0 WT. * and ** indicate statistically significant differences at *p* < .05 and *p* < .01, respectively. In this part, the experiments were done in three independent biological replicates and similar results were obtained. (h) Cluster analysis of differentially expressed genes (DEGs) in miR472 OE lines (miR472‐OE‐1# and miR472‐OE‐7#) and Col‐0 WT on the expression profiles measured by RNA‐Seq. (i) Manhattan (MA) plot of the DEGs between miR472 OE lines (miR472‐OE‐1# and miR472‐OE‐7#) and Col‐0 WT. (j) Volcano plot of the DEGs between miR472 OE lines (miR472‐OE‐1# and miR472‐OE‐7#) and Col‐0 WT. (k) GO classifications of DEGs across one comparison between miR472 OE lines (miR472‐OE‐1# and miR472‐OE‐7#) and Col‐0 WT. The *x* axis represents the number of DEGs in a category. The results of miR472 OE lines (miR472‐OE‐1# and miR472‐OE‐7#) versus Col‐0 WT are summarized in three main categories: molecular function (red), cellular component (green), and biological process (blue). (l) KEGG of the annotated DEGs across one comparison between miR472 OE lines (miR472‐OE‐1# and miR472‐OE‐7#) and Col‐0 WT. The *y* axis indicates the KEGG pathway. The *x* axis indicates the Rich factor. High *q* values are shown in red and low *q* values are shown in green

To examine the role of miR472 in plant immunity, and to determine whether miR472 is required in AR156‐triggered ISR, we generated transgenic plants with overexpression or knockdown/out of miR472. We hypothesized that if miR472 indeed modulates AR156‐triggered ISR, enhancement or suppression of miR472 expression would result in an altered defence response. The schematic diagram for our transgenic plants, successfully modified to overexpress *miR472* using the cauliflower mosaic virus (CaMV) 35S promoter, is shown in Figure 2a. miR472 overexpression had no major effects on the development of transgenic lines, but slightly altered growth was observed in transgenic plants (Figure 2b). Northern blotting showed that miR472 was expressed at high levels in the homozygous miR472‐overexpression (OE) lines (1#, 7#) (Figure 2c), which were subsequently used to analyse disease resistance and ISR. As expected, the miR472‐OE line was more susceptible to infection with Pst DC3000. Bacterial growth of Pst DC3000 in *Arabidopsis* leaves was determined at 0, 3, and 4 days post‐inoculation (dpi). The miR472‐OE lines (1#, 7#) showed a significantly higher growth of Pst DC3000 compared with that in wild‐type plants (Figure 2d). miR472 has previously been shown to negatively regulate PTI and ETI (Boccara *et al.*, 2014), therefore Pst DC3000 (*AvrPphB*) (Pst DC3000 expressing AvrPphB, which activates a strong ETI) and Pst DC3000 (*hrcC*) (type III secretion system [TTSS] mutant of Pst DC3000, which activates PTI) were used to test resistance to disease in *Arabidopsis*. The growth of Pst DC3000 (*AvrPphB*) and Pst DC3000 (*hrcC*) was determined at 0, 3, and 4 dpi. Our results indicate that Pst DC3000 (*AvrPphB*) and Pst DC3000 (*hrcC*) showed high levels of proliferation in the miR472‐OE lines (1#, 7#) (Figure 2e,f). These results agree with those of previous studies (Boccara *et al.*, 2014), and indicate that miR472 may extensively participate in PTI and ETI in *Arabidopsis*. We then evaluated the effects of miR472 on AR156‐induced systemic resistance in *Arabidopsis*. Compared with their respective controls, wild‐type Col‐0 plants pretreated with AR156 showed a significant (*p* < .05) reduction in disease severity, while the response of the miR472‐OE lines (1#, 7#) did not show a statistically significant difference (Figure 2g). These results indicate that miR472 may be involved in AR156‐triggered ISR.

**FIGURE 3:**
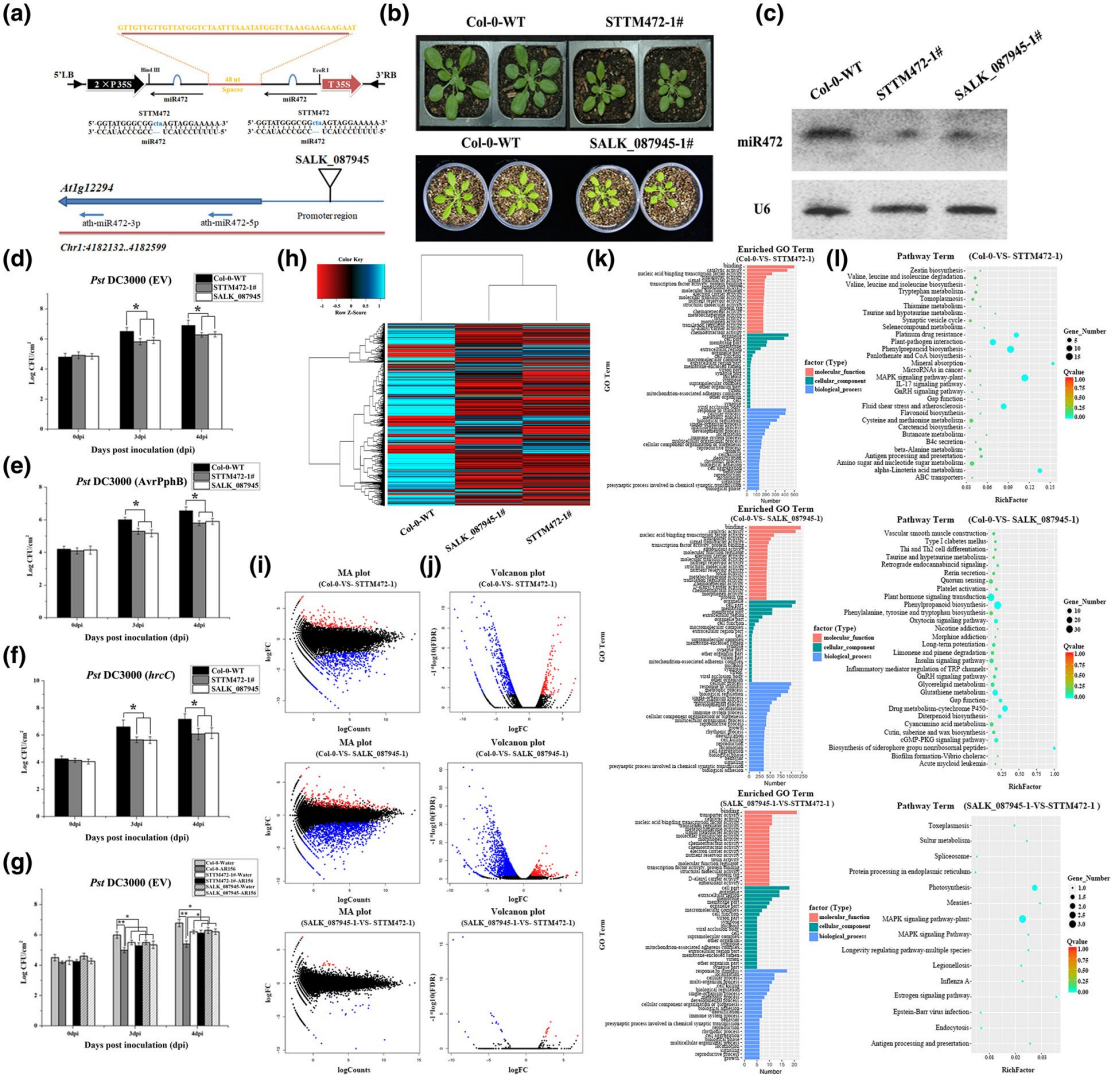
The miR472 knockdown and knockout showed more resistance to virulent *Pseudomonas syringae* pv. *tomato* (Pst) DC3000. The pathogen assay and induced systemic resistance (ISR) triggered by *Bacillus cereus* AR156 in *Arabidopsis* ecotype Col‐0 wild‐type (WT), miR472 knockdown lines (STTM472‐1#), and knockout lines (SALK_087945) were as done described in the materials and methods section. (a) Schematic diagram of the miR472 coding gene *At1g12294* structure and the STTM472 structure showing the design strategy. The STTM472 construct with miR472 binding sites on flanking sides separated by a 48‐nucleotide spacer (orange) forming an imperfect weak stem‐loop. The trinucleotide bulge sequences in the miRNA binding sites are shown in blue; the complementary miR472 is shown in black. nt, nucleotides; 2 × P35S, enhanced 35S promoter; T35S, terminator. For the miR472 coding gene structure, the triangle indicates the T‐DNA insertion position. (b) Phenotype of miR472 knockdown lines (STTM472‐1#) and knockout lines (SALK_087945) were compared to ecotype Col‐0 WT, and the images show the 4‐week‐old seedlings. (c) Expression of miR472 in the miR472 knockdown lines (STTM472‐1#) and knockout lines (SALK_087945) by northern blotting with an miR472 probe; U6 was used as a loading control. (d)–(f) Bacterial growth in 5‐week‐old plants from miR472 knockdown lines (STTM472‐1#), knockout lines (SALK_087945), and Col‐0 WT infiltrated with Pst DC3000 (EV) (OD_600_ = 0.0001), Pst DC3000 (*AvrPphB*) (OD_600_ = 0.0005), and Pst DC3000 (*hrcC*) (OD_600_ = 0.0001), respectively. (g) Bacterial growth assay of Pst DC3000 (EV) in miR472 knockdown lines (STTM472‐1#), knockout lines (SALK_087945), and Col‐0 WT pretreated with AR156 or 0.85% NaCl. For (d)–(g) the data are means and *SD* (*n* = 24). The LSD test was performed to determine the significant differences between miR472 knockdown/out lines and Col‐0 WT. * and ** indicate statistically significant differences at *p* < .05 and *p* < .01, respectively. In this part, the experiments were done in three independent biological replicates and similar results were obtained. (h) Cluster analysis of differentially expressed genes (DEGs) in miR472 knockdown/out lines and Col‐0 WT on the expression profiles measured by RNA‐Seq. (i) Manhattan (MA) plot of the DEGs between the miR472 knockdown/out lines and Col‐0 WT. (j) Volcano plot of the DEGs between the miR472 knockdown/out lines and Col‐0 WT. (k) GO classifications of DEGs across one comparison between the miR472 knockdown/out lines and Col‐0 WT. The *x* axis represents the number of DEGs in a category. The results of miR472 knockdown/out lines versus Col‐0 WT are summarized in three main categories: molecular function (red), cellular component (green), and biological process (blue). (l) KEGG of the annotated DEGs across one comparison between the miR472 knockdown/out lines and Col‐0 WT. The *y* axis indicates the KEGG pathway. The *x* axis indicates the Rich factor. High *q* values are shown in red and low *q* values are shown in green

To further investigate the role of miR472 in AR156‐triggered ISR, we generated transgenic lines of *Arabidopsis* bearing a knockdown or knockout of miR472 (Figure 3a). As shown in Figure 3b, the stature of the two types of transgenic plants was smaller than that of the wild‐type plant. Moreover, the expression of miR472, assessed using northern blotting, was significantly decreased in the transgenic line STTM472‐1# and T‐DNA insertion line SALK_087945‐1# compared with that in the wild‐type control (Figure 3c). The mutants were also assessed for disease resistance and ISR. As expected, the STTM472‐1# and SALK_087945‐1# displayed enhanced resistance to Pst DC3000, Pst DC3000 (*AvrPphB*), and Pst DC3000 (*hrcC*) compared with that observed in wild‐type plants. The density of bacterial cells on the leaves of the two mutant plant lines was decreased significantly (*p* < .05) compared with that of the wild type (Figure 3d–f). These results indicate that miR472 can act as a negative regulator of disease resistance in plants. We also examined how AR156 affected pathogen proliferation on the leaves of plants with bearing a knockdown or knockout of miR472. The leaves of wild‐type Col‐0 plants pretreated with AR156 and inoculated with Pst DC3000 showed significantly decreased (*p* < .01) pathogen density at 3 and 4 dpi compared with that of respective controls inoculated with Pst DC3000 only; by contrast, the density of Pst DC3000 on the leaves of STTM472‐1# and SALK_087945‐1# remained constant irrespective of pretreatment with AR156 (Figure 3g). These results indicate that AR156‐triggered ISR was abolished in miR472 mutant lines.

Next, we employed a comparative transcriptomic analysis using the different miR472 transgenic lines miR472‐OE‐1#, miR472‐OE‐7#, STTM472‐1#, SALK_087945‐1#, and wild‐type plant Col‐0 to examine the function of miR472 in plant defence against pathogenic invasion. The libraries constructed using different types of *Arabidopsis* were sequenced using a HiSeq 2500 platform in BGI. Approximately 27.1 Gb of clean reads was obtained after rigorous quality checking. As shown in Tables S5 and S6, the Q20 percentage of each library reached 96%, the Q30 percentage of each library was in the range 91.0%–92.2%, the clean reads ratio percentage of each library reached 99%, and the total mapping ratio was in the range 94.5%–95.1% (Tables S5 and S6). We then compared RNA‐Seq data obtained using transgenic lines and wild‐type plants, with each assessment being performed four times (Figure S2a). Our results show that increases and decreases in the expression level of miR472 induced changes in the expression of numerous plant genes (Table S7 and Figure S1); 2,118 DEGs showed significant changes in expression (1,521 down‐regulated and 597 up‐regulated) when we compared expression profiles of wild‐type Col‐0 and transgenic miR472‐OE‐1# and miR472‐OE‐7#. Additionally, 1,855 (1,464 down‐regulated and 391 up‐regulated) and 732 (561 down‐regulated and 171 up‐regulated) DEGs with significant changes in expression levels were identified when we compared expression profiles among wild‐type Col‐0, STTM472‐1#, and SALK_087945‐1# (Figure S1a). The fold changes in the expression of these DEGs are shown in a heat map in Figures 2h and 3h. The Manhattan and volcano plots also show the relationship between fold‐changes and miR472 overexpression in plant lines with a knockdown/out of miR472 and wild‐type plants (Figures 2i,j and 3i,j). In summary, these results indicate that differential expression of miR472 altered the expression of numerous plant genes. Moreover, analysis of the DEGs in miR472‐OE‐1# versus Col‐0, miR472‐OE‐7# versus Col‐0, STTM472‐1# versus Col‐0, and SALK_087945‐1# versus Col‐0, performed using a Venn diagram, identified 503 overlapping genes among the three groups (Figure S1b). This result indicates that these genes may be associated with miR472 regulation and expressed specifically during miR472‐regulated immune response. Otherwise, to gain insight into the potential biological processes associated with miR472 regulation process in which DEGs were involved, we performed analysis of GO and KEGG pathways. Comparison of Col‐0 and miR472‐OE lines showed that showed that 22 GO terms corresponded with “cellular component”, 20 with “molecular function”, and 93 with “biological process”. Moreover, the terms “organelle” and “cell part” were most enriched in the term “cellular component”. “Binding” was the most frequent term in “molecular function” followed by “catalytic activity” and “nucleic acid binding transcription” (Figure 2k). Furthermore, in comparison with Col‐0 and STTM472‐1# or SALK_087945‐1#, respectively, in total, 141 and 240 GO terms were assigned and significantly enriched (*p* < .05) to the DEGs, respectively. For Col‐0 versus STTM472‐1#, 12 GO terms corresponded to “cellular component”, 22 GO terms to “molecular function”, and 107 GO terms to “biological process”; for Col‐0 versus SALK_087945‐1#, 18 GO terms corresponded to “cellular component”, 66 GO terms to “molecular function”, and 156 GO terms to “biological process” (Figure 3k). These results indicate that miR472 could change the physiological and biochemical functions in *Arabidopsis*. We next conducted an analysis of KEGG pathways assigned to DEGs, and our results show that the top 30 pathways were significantly enriched (*p* < .05) (Figures 2l and 3l). Among these pathways, the MAPK signalling pathway, plant hormone signalling transduction pathway, and several others were related to disease resistance. These results confirm that miR472 is involved in plant defence response.

### 2.3 miR472 alters defence response to Pst DC3000 by targeting a group of plant resistance genes in *Arabidopsis*

**FIGURE 4:**
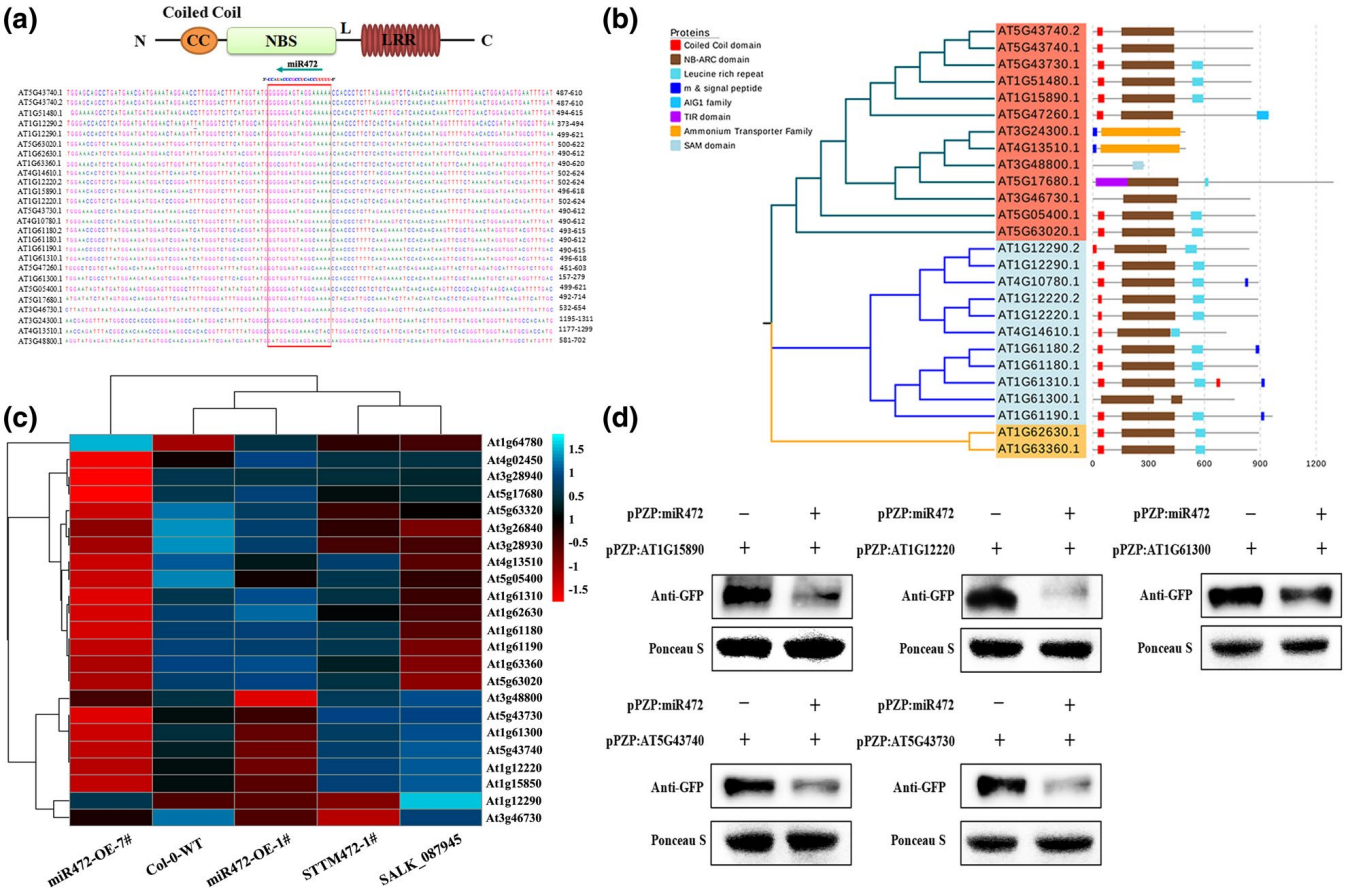
The prediction and validation of miR472 target genes. (a) The predicted information of miR472 target genes. Nucleotide sequences targeted by miR472 were aligned to corresponding genes, with the CC‐NBS‐LRR gene structure depicted at the top. CC, coiled‐coil domain; NBS, nucleotide‐binding site domain; LRR, leucine‐rich repeat domain. Red boxes on gene sequences represent the target binding site by miR472. (b) Phylogenetic tree of predicted targets based on the amino acid sequence. Sequences were aligned using ClustalX, and the tree was generated using the online software EvolView v. 3 (Subramanian *et al.*, 2019). (c) Cluster analysis of predicted miR472 target genes in miR472 overexpression lines, knockdown/out lines and *Arabidopsis thaliana* Col‐0 wild‐type (WT) on the expression profiles measured by RNA‐Seq. (d) Expression levels of miR472 targets fused with green fluorescent protein (GFP) in *Nicotiana benthamiana* by western blotting with a commercial GFP antibody that crossreacts with GFP tag. Ponceau S staining was used as protein loading control. The experiments were done in three independent biological replicates and similar results were obtained

**FIGURE 5:**
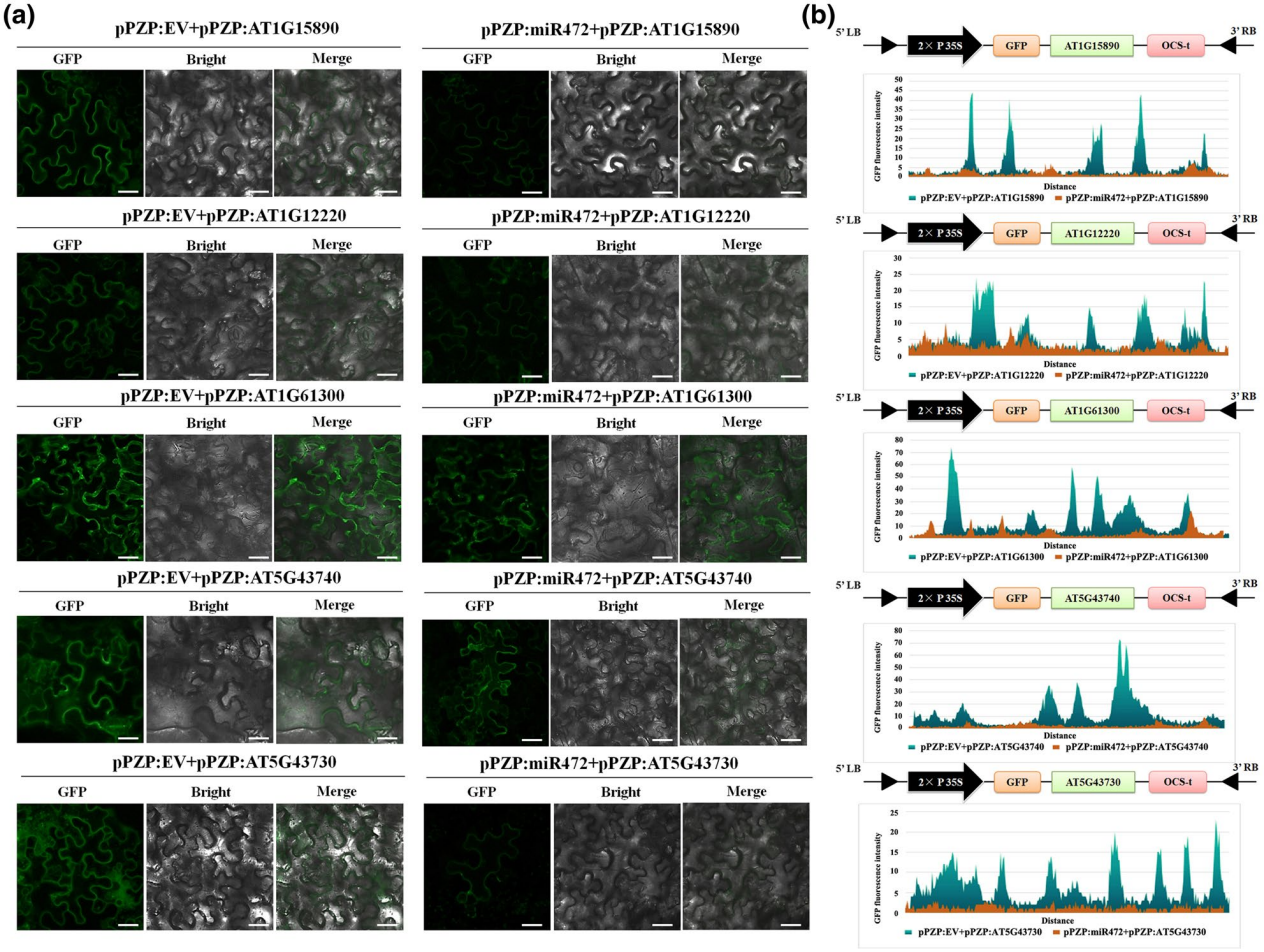
Five targets of miR472 were certificated by green fluorescent protein (GFP) fluorescence fusion labelling in vitro. Confocal imaging of *Nicotiana benthamiana* epidermal cells expressing miR472 and its targets. (a) Epidermal cells of *N. benthamiana* leaves transiently expressing miR472 target coding genes alone, when fused with a GFP tag or co‐expressing with miR472 were observed using confocal microscopy and the photographs were taken 48 hr after infiltration. Scale bars = 20 μm. (b) The expression levels of target genes were quantified through detecting the fluorescence intensity. The values on the *y* axis indicate the relative intensity of the GFP fluorescence signal. The experiments were done in three independent biological replicates and similar results were obtained

miRNAs regulate post‐transcriptional gene expression by mRNA degradation and/or translational repression, therefore identification of miRNAs targets is important to determine the mechanisms involved in miRNA activity. As previously shown, miR472 targets dozens of CC‐NB‐LRRs (CNLs) to initiate the production of RDR6‐dependent secondary siRNAs (Boccara *et al.*, 2014). Therefore, we next aimed to find and validate more targets of miR472, and to determine which of these targets are involved in AR156‐triggered ISR. In total, 37 targets were predicted by the plant small RNA target analysis server, psRNATarget; 26 of these targets were CNLs (Table S8). In Figure 4a, the large red box indicates the predicted target region of miR472 in CNLs. Moreover, phylogenetic analyses based on amino acid sequences of the 26 predicted CNLs indicated the presence of three groups (Figure 4b). Subsequently, we analysed the differential expression of 26 predicted targets, as well as RNA‐Seq data. As shown in Figure 4c and Table S9, 20 CNLs were down‐regulated in miR472‐OE‐1# and miR472‐OE‐7#, and seven CNLs were up‐regulated in both STTM472‐1# and SALK_087945‐1# compared with the profile of wild‐type Col‐0 plants. Finally, five CNLs identified as possible targets of miR472 were identified as *At1g15890*, *At1g61300*, *At1g12220*, *At5g43730*, and *At5g43740*. To confirm the transcriptomic data, expression of these five CNLs was analysed by quantitative reverse transcription PCR (RT‐qPCR), which indicated that the patterns of gene expression in these five CNLs were consistent with those shown by RNA‐Seq (Figure S2). To confirm that these five genes were indeed true targets of miR472, we performed *Agrobacterium*‐mediated transient co‐expression in *Nicotiana benthamiana* to express green fluorescent protein (GFP):CNL fusion protein alone and co‐expressing miR472*.* The results of western blotting indicate that co‐expression of miR472 with the five CNLs significantly decreased the mRNA levels of the CNLs (Figure 4d), which was not observed in the case of the GFP:CNLs fusion protein. Confocal imaging of *N. benthamiana* epidermal cells revealed that accumulation of CNLs in the cells was suppressed via co‐expression with miR472 (Figure 5a). Moreover, analysis of fluorescence intensity showed that co‐expression of miR472 with the five CNLs significantly decreased accumulation of GFP compared with the levels of respective controls (Figure 5b). We then performed *Agrobacterium*‐mediated transient co‐expression assays to express a β‐glucuronidase (GUS):CNL fusion protein alone and in co‐expression with miR472 in *N. benthamiana*. GUS histochemical staining was used to verify the results. As shown in Figure S3, the results of GUS histochemical staining indicated that GUS expression decreased significantly in the leaves of plants in which miR472 was co‐expressed with the five CNLs (Figure S3). So far, these findings indicate that these five CNLs are the true targets of miR472.

**FIGURE 6:**
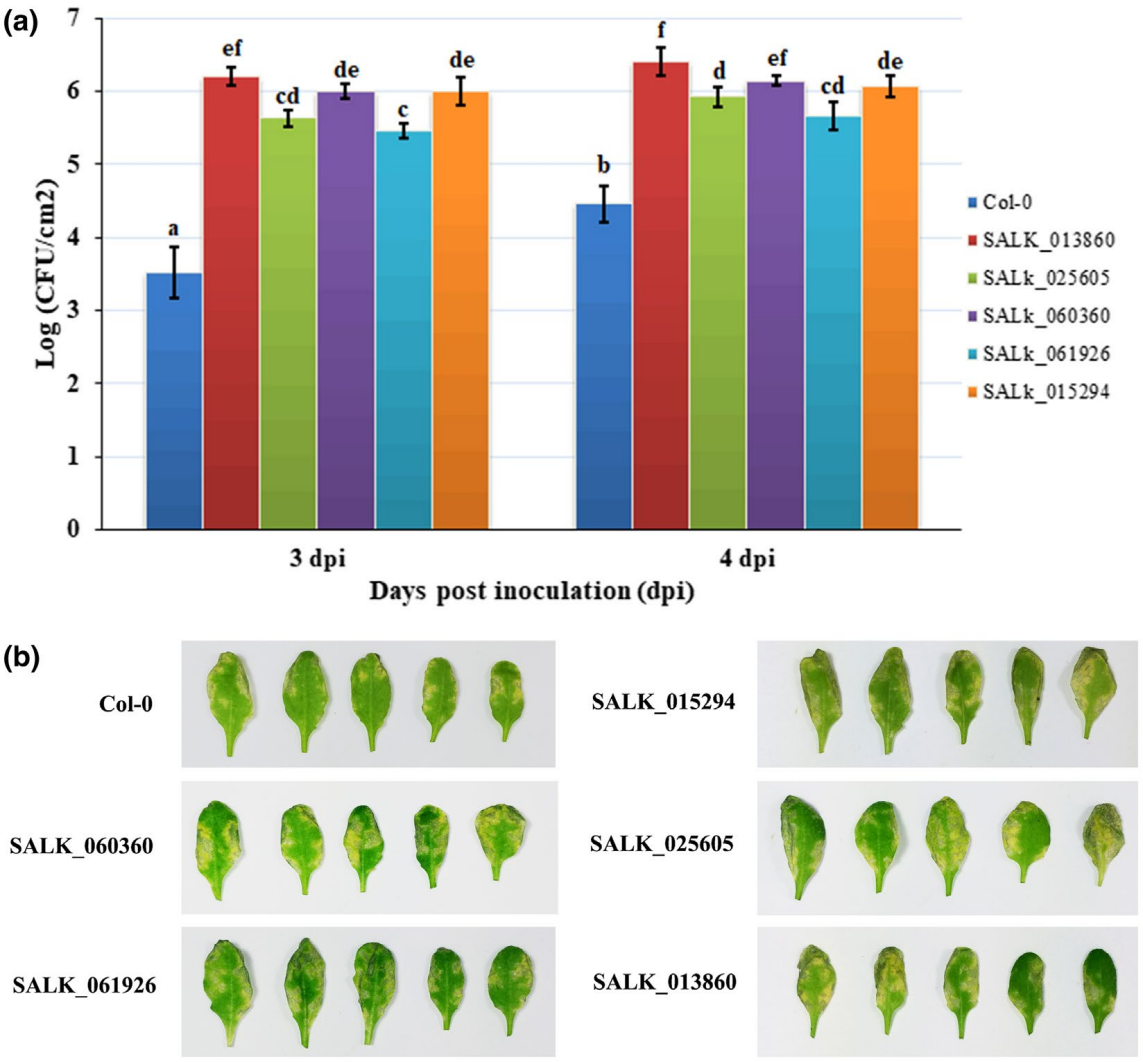
Reduced basal resistance against virulent *Pseudomonas syringae* pv. *tomato* (Pst) DC3000 observed in the miR472 target genes mutant lines. (a) Bacterial growth in 5‐week‐old *Arabidopsis thaliana* plants from T‐DNA insert lines of miR472 target genes and Col‐0 wild‐type (WT) infiltrated with Pst DC3000 (EV) (OD_600_ = 0.0001), the T‐DNA insert lines being SALK_060360C (*At1g15890*), SALK_061926C (*At1g61300*), SALK_015294C (*At1g12220*), SALK_025605C (*At5g43740*), and SALK_013860C (*At5g43730*). The data are means and *SD* (*n* = 24). The LSD test was performed to determine the significant differences between T‐DNA insert lines and Col‐0 wild‐type (WT). Letters above the bars indicate statistically significant differences between treatments (LSD test, *p* < .05). (b) Disease symptoms caused by Pst DC3000 (empty vector, EV) infection in different T‐DNA insert lines and Col‐0 WT plants. A representative plant from each treatment was photographed at 5 days post‐inoculation. All the experiments were done in three independent biological replicates and similar results were obtained

We evaluated the role of these five CNLs in plant defence response to Pst DC3000. For this, we generated transgenic lines bearing a knockout of these five CNLs and used these lines for a pathogen challenge assay. The five T‐DNA insertion mutant lines, obtained from TAIR, were designated as SALK_060360C, SALK_061926C, SALK_015294C, SALK_025605C, and SALK_013860C, and contained T‐DNA insertions in *At1g15890*, *At1g61300*, *At1g12220*, *At5g43740*, and *At5g43730*, respectively. On challenge with Pst DC3000, all five CNL lines exhibited significantly higher bacterial growth (Figure 6a). Compared with Col‐0 wild‐type plants, the five CNL lines showed increased susceptibility to disease, which was manifested as stronger yellowing or chlorosis in leaves (Figure 6b). These results indicate that the five identified targets of miR472 positively regulate disease resistance, thereby further clarifying the role of miR472 in regulating the defence response.

### 2.4 Target genes of miR472 are coordinately expressed with *B. cereus* AR156‐triggered ISR

**FIGURE 7:**
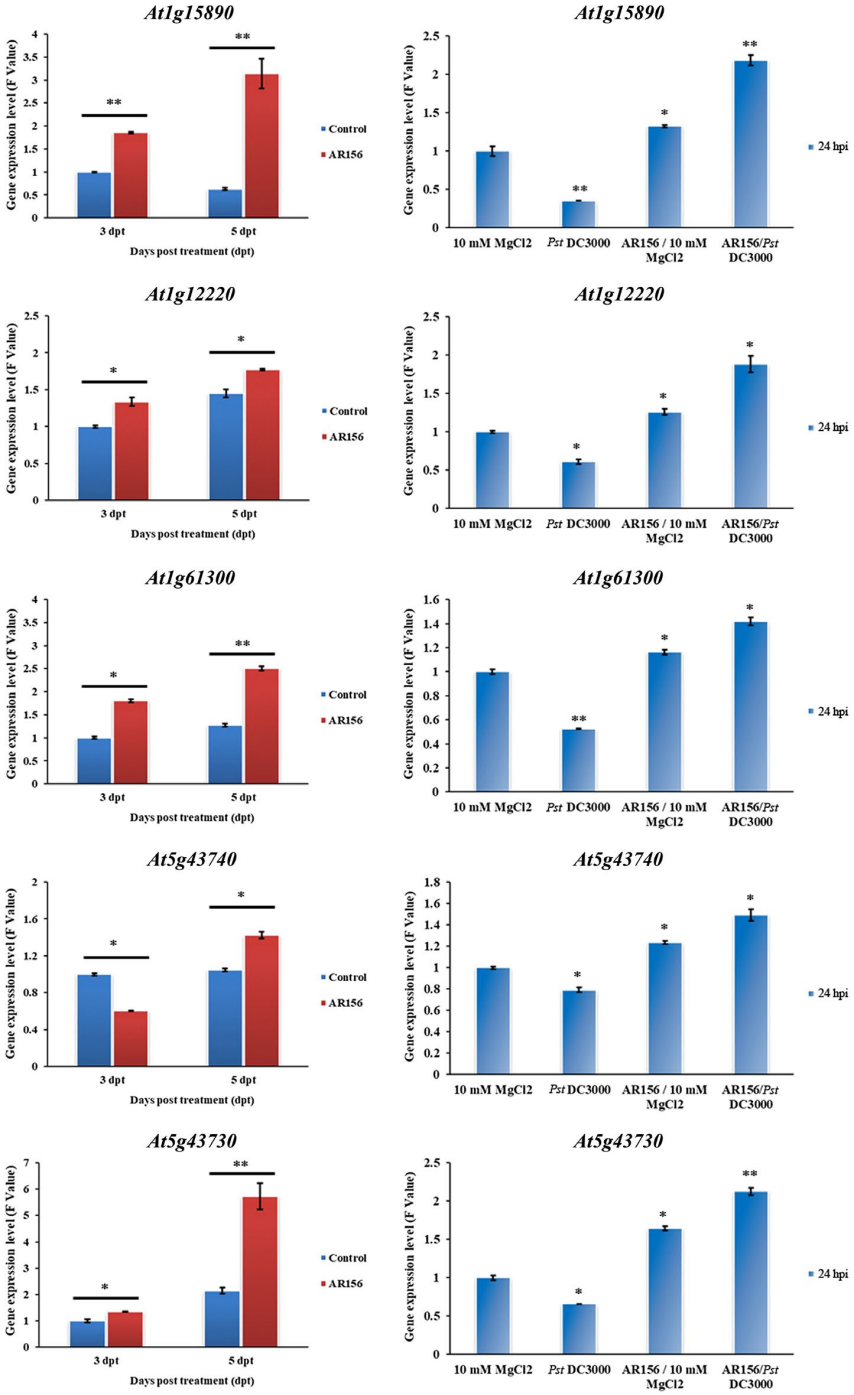
Target genes of miR472 were coordinately expressed with *Bacillus cereus* AR156‐triggered induce systemic resistance (ISR). The expression profiles of miR472 target genes *At1g15890*, *At1g12220*, *At1g61300*, *At5g43740*, and *At5g43730* in *Arabidopsis thaliana* seedlings with AR156‐triggered ISR were detected. The plants were pretreated with AR156 at 5 × 10^7^ cfu/ml for 5 days and 0.85% NaCl. Subsequently, leaves were spray inoculated with *Pseudomonas syringae* pv. *tomato* (Pst) DC3000 (empty vector, EV) (OD_600_ = 0.1). The leaves in different treatments were harvested at different time points and total RNA was extracted (dpt, days post‐treatment; hpi, hours post‐inoculation). Expressions of miR472 target genes were examined by quantitative reverse transcription PCR. The expression values of the individual genes were normalized using *β‐Tubulin 4* as an internal standard. The data are means and *SD* (*n* = 24). The LSD test was performed to determine the significant differences between T‐DNA insert lines and *A. thaliana* Col‐0 wild‐type. * and ** indicate statistically significant differences at *p* < .05 and *p* < .01, respectively. The experiments were done in three independent biological replicates and similar results were obtained

Subsequently, we examined whether miR472 may modulate AR156‐triggered ISR by regulating its five putative CNL targets. For this we used RT‐qPCR to analyse the expression levels of these five CNLs during AR156‐triggered ISR. Our results show that all five targets showed significantly altered expression levels in AR156‐treated *Arabidopsis* compared with mock‐treated controls; this expression pattern was not dependent on inoculation with Pst DC3000. As shown in Figure 7, the expression of *At1g15890*, *At1g61300*, *At1g12220*, *At5g43740*, and *At5g43730* was up‐regulated in the leaves of plants treated only with AR156 for 3 and 5 days compared with that of plants administered mock treatment. These target genes were also up‐regulated in plants pretreated with AR156 and then challenged with Pst DC3000. Increases in the expression of these genes were significantly greater compared with those in plants treated only with Pst DC3000 (Figure 7). In summary, our results indicate that the target genes of miR472 were coordinately expressed and involved in the modulation of AR156‐triggered ISR against the invasion of Pst DC3000.

## 3 DISCUSSION

### 3.1 Plant endogenous small RNA miR472 plays an essential role in *B. cereus* AR156‐triggered ISR

ISR triggered by PGPRs is an important mechanism in broad‐spectrum biocontrol against various pathogens. The molecular mechanisms underlying ISR have been studied extensively in various plant species such as *A. thaliana*. Many signalling components and molecular processes are involved in the interactions between ISR elicitors and the host (Pieterse *et al.*, 2014). For instance, *B. cereus* AR156 can trigger ISR by simultaneously activating the SA and JA/ET signalling pathways in an NPR1‐dependent manner and the transcription factors WRKY70 and WRKY11 were identified as “biological switches” involved in the regulation of these processes (Niu *et al.*, 2011; Jiang *et al.*, 2016). Furthermore, the regulatory mechanisms involved in PGPR‐triggered ISR are gradually being revealed. Small RNAs in plants are important regulators of plant defence against pathogens (Huang *et al.*, 2016; Niu *et al.*, 2016). miRNA can negatively regulate the expression of plant R genes (Zhai *et al.*, 2011; Li *et al.*, 2012; Shivaprasad *et al.*, 2012). The expression of miR398, miR482, and miR5300 is increased by targeting a group of R genes via challenge with *F. oxysporum* in resistant tomato cultivars (Ouyang *et al.*, 2014). As for small RNAs involving in regulating PGPR‐triggered ISR, we reported that expression of the miR825/825* pair was significantly down‐regulated by infection with Pst DC3000 in plants pretreated with AR156 (Niu *et al.*, 2016). We used small RNA deep sequencing data obtained previously (Niu *et al.*, 2016) to seek more small plant RNAs participating in AR156‐triggered ISR systemically, and to further explore how they regulate the process of AR156‐triggered ISR in this study. For this, we evaluated the differential expression levels and function of target genes such as transcription factors and R genes. Our results indicate that the expression of miR472 was significantly down‐regulated by pretreatment with AR156 and challenged with Pst DC3000, while it was slightly induced by Pst DC3000 infection only (Figure 1c–e). Northern blotting confirmed that accumulation of miR472 in *Arabidopsis* was modulated by *B. cereus* AR156 (Figure 1f). The effect of miR472 on AR156‐triggered ISR was also analysed using plant lines with miR472 knockdown and knockout. As shown in Figure 2g, treatment with AR156 significantly reduced (*p* < .05) the density of Pst DC3000 in the leaves of wild‐type Col‐0 at 3 and 4 dpi compared with that on the leaves of respective controls. However, AR156‐induced reduction of Pst DC3000 density in the leaves of the miR472‐OE lines (1#, 7#) was not significant (*p* < .05) (Figure 2g). Conversely, the density of Pst DC3000 in the leaves of miR472 mutant lines STTM472‐1# and SALK_087945‐1# remained constant with or without AR156 treatment (Figure 3g). These findings indicate that miR472 plays an essential role in AR156‐triggered ISR. In future studies we will investigate whether this miR472‐mediated pathway represents a new mechanism in ISR regulation.

### 3.2 *Arabidopsis* miR472 negatively regulates PTI and ETI responses to the infection of Pst DC3000

miR472‐mediated post‐transcriptional regulation was found to be involved in plant defensive responses against Pst DC3000. Boccara *et al*. discovered that miR472 could target several NBS‐LRR genes in an RDR6/DCL4‐dependent manner. The targeting of mRNA by 22‐nt miRNAs was assisted by a cascade of secondary siRNAs that were generated in an RDR6‐dependent manner (Boccara *et al.*, 2014). In our present study, it was found that the miR472‐OE lines (1#, 7#) were more susceptible to infection with Pst DC3000, while plants with knockdown and knockout of miR472 were more resistant to this pathogen. The miR472‐OE lines (1#, 7#) displayed a significantly higher growth of Pst DC3000 compared with that of wild‐type plants; however, the growth of Pst DC3000 declined sharply in miR472 mutant lines STTM472‐1# and SALK_087945‐1# (Figures 2d and 3d). miR472 negatively regulated PTI responses to the infection of Pst DC3000. Boccara *et al.* found that the induction of RSG1and RPS5 mRNA via flg22 was significantly impaired in miR472‐OE lines (1#, 7#) compared with that in wild‐type controls. However, production of reactive oxygen species triggered by flg22 remained normal, while callose deposition was reduced in miR472‐OE lines (1#, 7#) compared with that in wild‐type controls (Boccara *et al.*, 2014). In our present study, the miR472 transgenic plants were inoculated with Pst DC3000 (*hrcC*), and the growth of Pst DC3000 (*hrcC*) was then determined in *Arabidopsis* leaves at 0, 3, and 4 dpi. We found that Pst DC3000 (*hrcC*) showed high growth levels in the miR472‐OE lines (1#, 7#), but low growth levels in the miR472 mutant lines STTM472‐1# and SALK_087945‐1#, compared with that in wild‐type control (Figures 2f and 3f). These results indicate that miR472 is involved in the regulation of the PTI process.

Plants have evolved an extensive repertoire of immune receptors, and R proteins monitor vital host proteins for signs of pathogenic activity. miR472 and RDR6‐dependent secondary siRNAs are involved in RPS5‐mediated resistance (Simonich and Innes, 1995; Dangl and Jones, 2001; Boccara *et al.*, 2014). In our present study, we also observed that Pst DC3000 (*AvrPphB*) multiplied to high levels in the miR472‐OE lines (1#, 7#), but remained at low levels in the miR472 mutant lines STTM472‐1# and SALK_087945‐1# compared with the levels observed in the wild‐type plant (Figures 2e and 3e). Taken together, we proved that miR472 is involved in both ETI and PTI via the use of mutants of Pst DC3000 that activate different branches of plant immune system. Although these results were consistent with those obtained in previous studies (Boccara *et al.*, 2014), our approach and methodology differed.

### 3.3 An altered expression level of miR472 activates a large number of DEGs

Transcriptome sequencing is a widely used, highly efficient, molecular biological research method used to obtain detailed transcriptome information (Chen *et al.*, 2017; Jiang *et al.*, 2019). To further detail how miR472 regulates plant disease resistance, we used the transgenic plants miR472‐OE‐1#, miR472‐OE‐7#, STTM472‐1#, SALK_087945‐1#, and wild‐type Col‐0 for transcriptome sequencing. In this study, the results inform us that an altered expression level of miR472, regardless of whether it was enhanced or decreased, activated a large number of DEGs (Table S7 and Figure S1). Thousands of DEGs were identified in miR472 transgenic plants compared with the profile of wild‐type plant Col‐0 (Figures 2h–j and 3h–j). However, only a few DEGs were identified in data generated using STTM472‐1# and SALK_087945‐1# (Figure 3h–j). The comparison of miR472 knockdown line STTM472‐1# and knockout line SALK_087945‐1# indicates that miR472 mutants were generated successfully and were a reliable model for analysis using transcriptome sequencing. MAPKs are involved in plant growth, development, and interactions between plants and pathogens (Yu and Tang, 2004; Bi and Zhou, 2017). MAPKs also play important roles in plant response to pathogen infections, and many pathogens secrete effectors that can inhibit the MAPK cascade (Bi and Zhou, 2017). In our present study, we also analysed the GO and KEGG pathways, and found that miR472 was involved in the MAPK signalling pathway that responds to plant pathogens (Figures 2k,l and 3k,l). Here, by using transcriptome analysis techniques, we discovered that endogenous miR472 plays an important role in PTI mediated by the MAPK cascade.

### 3.4 miR472 modulates the defence response to Pst DC3000 by targeting a group of CNLs in *Arabidopsis*

miRNAs can regulate the plant defence response by targeting numerous R genes. In our study, 37 genes were identified as targets of miR472 via bioinformatics prediction. Twenty‐six of these genes were CNLs and included the previously reported targets *At1g12220*, *At1g51480*, and *At5g43730* (Table S8; Boccara *et al.*, 2014). Subsequently, the five CNLs *At1g15890*, *At1g61300*, *At1g12220*, *At5g43730*, and *At5g43740* were identified as possible targets of miR472 via RNA‐Seq and RT‐qPCR validation (Figures 4c and S2, and Table S9). To confirm the five identified targets, we also performed *Agrobacterium*‐mediated transient co‐expression assays. Using western blotting, GFP fluorescence, and GUS histochemical staining, we found that miR472 can inhibit the accumulation of proteins encoded by these five target genes (Figures 4d, 5, and S3). To clarify the role of these five CNLs in the defence response to Pst DC3000, we generated transgenic lines bearing a knockout of these five CNLs and used them for a pathogen challenge with Pst DC3000. Following infection with Pst DC3000, all five CNL T‐DNA insertion lines showed significantly higher pathogenic bacterial growth (Figure 6a). Compared with that of wild‐type Col‐0 plants, all five CNL mutants showed increased susceptibility to disease, manifested as stronger yellowing or chlorosis of the leaves (Figure 6b). These results show that the five identified targets of miR472 positively regulated disease resistance in *Arabidopsis*. We also identified four new CNL genes that were targeted by miR472 and could mediate plant defence response. Our results confirm that miR472‐mediated plant defence depends not only on RPS5‐mediated ETI, but also on other CNLs. This finding also complements previous studies reported by Boccara *et al.* (2014).

### 3.5 *B. cereus* AR156‐triggered ISR against Pst DC3000 depends on CNL‐mediated basal immunity in *Arabidopsis*

Studies examining the molecular mechanisms of ISR have mainly focused on the roles of the crucial signalling pathways and key regulated proteins. Pieterse *et al*. demonstrated that WCS417r‐triggered ISR was dependent on the JA/ET signalling pathway and NPR1 in *Arabidopsis* (Pieterse *et al.*, 1996). In our previous study, we found that AR156 triggered ISR by simultaneously activating the SA and JA/ET signalling pathways in a manner dependent on the expression of the *NPR1* gene, and the transcription factors WRKY70 and WRKY11 were identified as “biological switches” involved in the regulation of these processes (Niu *et al.*, 2011; Jiang *et al.*, 2016). In this study, we wanted to examine whether miR472 is also involved in AR156‐triggered ISR. For this, we used RT‐qPCR to analyse the expression of miR472 targets during the process of AR156‐triggered ISR. Our results revealed that all of the targets showed significantly altered expression levels in AR156‐treated *Arabidopsis* compared with those in mock‐treated seedlings; this pattern of expression was not dependent on inoculation with Pst DC3000. The target genes of miR472 were up‐regulated in plants pretreated with AR156 and challenged with Pst DC3000. Increases in the expression of these genes were significantly greater than those in plants treated with Pst DC3000 alone (Figure 7). These findings indicate that miR472 performs a regulatory function in AR156‐triggered ISR by targeting of the expression levels of CNL genes involved in disease resistance. However, how these CNLs mediate plant immunity and ISR remains to be detailed in our future studies.

### 3.6 A proposed model for miR472 functions in regulating AR156‐triggered ISR

**FIGURE 8:**
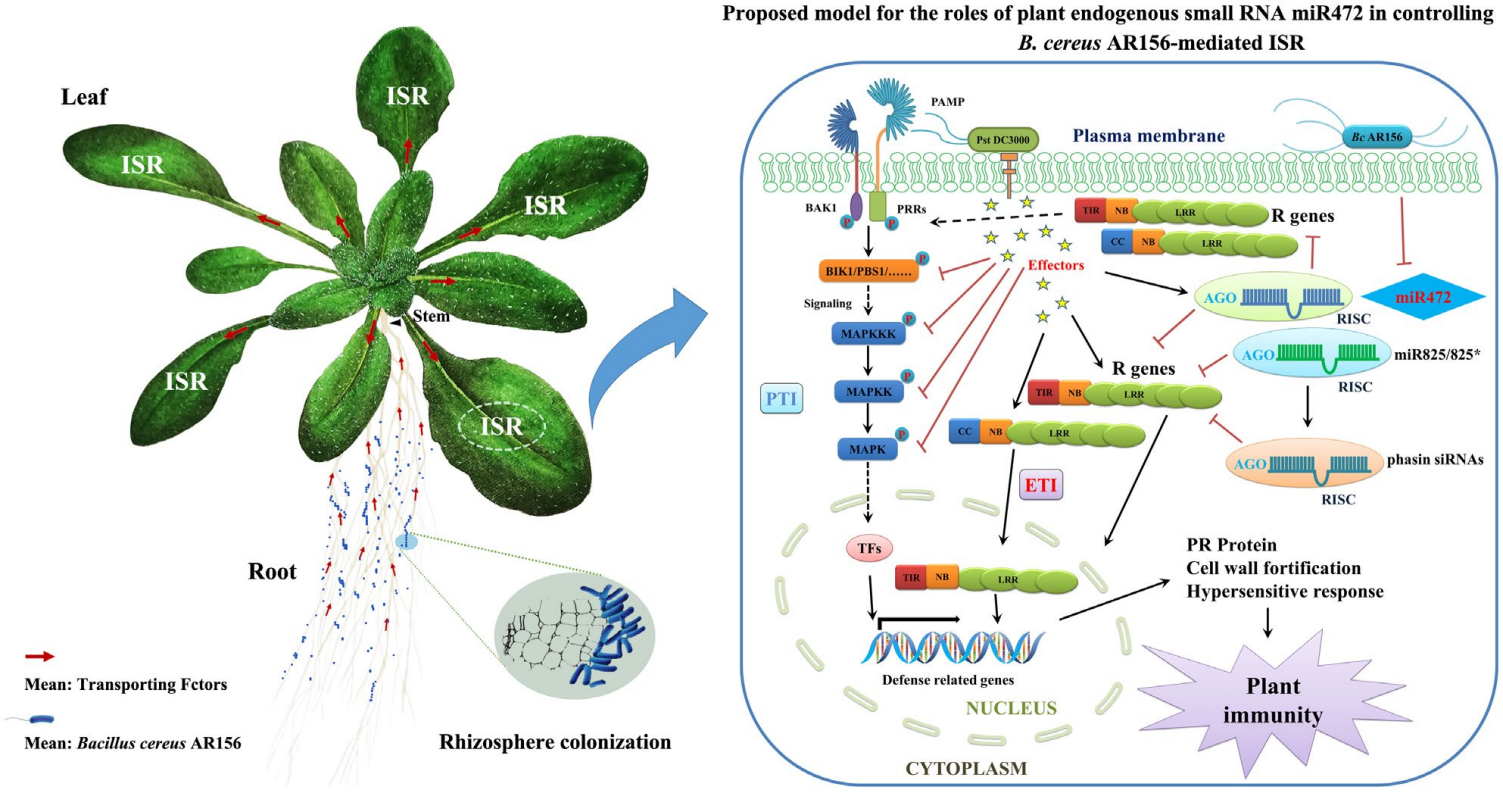
Proposed model for the roles of plant endogenous small RNA miR472 in controlling *Bacillus cereus* AR156‐mediated induced systemic resistance (ISR)

In summary, we identified miR472 as an important regulator of AR156‐triggered ISR. *Arabidopsis* roots and leaves were used to examine the regulatory mechanisms of endogenous small RNA in AR156‐triggered ISR (Figure 8). In *Arabidopsis*, MAMPs of *B. cereus* AR156 are recognized via their respective receptors, leading to the generation and release of a distinct ISR signal in the roots, which is then transferred to the leaves. To trigger ISR, AR156 suppresses the expression of miR472, which is involved in plant basal immunity, then increases the accumulation of proteins encoded by the CNL genes targeted by miR472. Therefore, the expression of R genes, involved in mediation of PTI and ETI, is considerably enhanced, thereby increasing the expression of PR proteins, activating hypersensitive responses, and increasing host resistance against pathogens such as Pst DC3000. In our present study, we determined how *B. cereus* AR156 triggers ISR by using miR472 to enhance the expression of R genes and increase the accumulation of their protein products.

To the best of our knowledge, this study is the first to determine that miR472 plays an important role in regulating PGPR‐triggered ISR in *Arabidopsis* via targeting five CNLs and, more importantly, provides a new way to study the mechanism of regulation on PGPR ISR to pathogens. Moreover, it provides theoretical guidance for the development and application of biopesticides.

## 4 EXPERIMENTAL PROCEDURES

### 4.1 Plants, bacterial strains, and growth conditions

All the plants used in this study are listed in Table S1. The seeds for each line were sown in vermiculite, then 3‐week‐old seedlings were transferred into 200‐ml pots filled with a mixture of sterilized vermiculite and potting soil at a volume ratio of 2:1, with one seedling per pot. The transplanted seedlings were cultivated in a growth chamber with a 10 hr day (200 μmol⋅m^−2^⋅s^−1^ at 22 °C) and a 14 hr night (20 °C) cycle at 70% relative humidity and supplied with modified half‐strength Hoagland nutrient solution (Hoagland and Arnon, 1938) weekly.

All the bacterial strains used in this study are listed in Table S2. The pathogens were grown in liquid King's B medium containing 50 mg/L rifampicin at 28 °C overnight. The cultured Pst DC3000 cells were pelleted by centrifugation at 5,000 × g for 10 min, and then resuspended in 10 mM MgSO_4_ containing 0.01% (vol/vol) of surfactant Silwet L‐77 (Sigma). Cell concentration was adjusted with respect to experimental purposes, using OD_600_ = 0.01 for spray inoculation and OD_600_ = 0.0001 for inoculation via syringe infiltration (as described by Jiang *et al.*, 2016) with several modifications; the concentrations of Pst DC3000 and Pst DC3000 (*hrc C*) were adjusted to OD_600_ = 0.0005 for use.

*B. cereus* AR156 was grown on Luria Bertani (LB) agar at 28 °C for 24 hr. Subsequently, the AR156 cells were pelleted by centrifugation at 5,000 × g for 10 min and resuspended in sterile 0.85% NaCl; cell concentration was adjusted to 5 × 10^7^ cfu/ml for experimental purposes.

### 4.2 Plant inoculations and bacterial counts

Six‐week‐old *Arabidopsis* seedlings were challenged with the indicated pathogens and then assessed for ISR using methods published previously (Katiyar‐Agarwal and Jin, 2007; Niu *et al.*, 2011; Jiang *et al.*, 2016). For the pathogen challenge procedure, we used a suspension containing Pst DC3000 (OD_600_ = 0.0001), which was inoculated via syringe infiltration. Leaf samples of each treated plant were collected by acock borer. Bacterial titres were determined by plating the organisms and counting the number of colonies at 0, 3, and 4 dpi. At least 24‐leaf discs were collected for each treatment. The density of Pst DC3000 in the *Arabidopsis* leaves was determined and expressed as cfu/g fresh leaf. For the ISR assay, 10 ml AR156 cell suspension (5 × 10^7^ cfu/ml) was poured onto the soil around the root of each seedling in each pot; an equivalent volume of 0.85% NaCl solution was used as control. Half of the seedlings receiving each treatment were challenged after 5 days by spraying the leaves with Pst DC3000 cell suspension (OD_600_ = 0.01) until all the *Arabidopsis* leaves were covered with fine droplets. The remaining seedlings were sprayed with 10 mM MgSO_4_ as control treatment. All the seedlings were maintained in a dew chamber at 100% relative humidity for 3 days and transferred to a growth chamber after inoculation. As described above, bacterial titres were determined at 0, 3, and 4 dpi. Each experiment was performed three times.

### 4.3 Genetic cloning, vector construction, and generation of transgenic plants

All the plasmids and primers used in this study are listed in Tables S3 and S4, respectively. To generate an miR472 overexpression construct, miR472 precursors were cloned into the Gateway destination vector pEG100 (Earley *et al.*, 2006). The miR472 knockdown vector, STTM‐472, used to inactivate miR472 in *Arabidopsis*, was generated according to the method of Yan *et al. *(2012). Next, we generated the overexpression construct of miR472 target genes according to methods described by Tzfira *et al. *(2005). Full‐length cDNA sequences of these R genes were cloned via PCR amplification. The PCR fragments of full‐length R‐protein coding sequences were inserted into the pPZP‐RCS1 vector (Tzfira *et al.*, 2005; Wang *et al.*, 2017).

All the constructs described above were electroporated into *Agrobacterium tumefaciens* GV3101 according to the methods described by Clough and Bent (1998). Generation of the transgenic *Arabidopsis* lines was performed using a floral dip method as reported previously (Koornneef and Pieterse, 2008); transformed seedlings were selected as described by Harrison *et al. *(2006) and at least two transgenic lines of each gene were selected.

### 4.4 RNA extraction, RT‐qPCR, and hybridization

For RNA extraction, 0.5 g fresh weight *Arabidopsis* leaves was collected. Total RNA for each sample was prepared using TRIzol (Invitrogen) as described in the manufacturer's recommendations. RT‐PCR was performed using 0.5 mg total RNA. Reverse transcription was conducted using HiScript Q Select RT Super Mix (Vazyme) (Jiang *et al.*, 2016). RT‐qPCR was conducted using a 7500 Real‐Time PCR System (ABI) with a SYBR Premix Ex Taq II (Takara) and 200 nM final primer concentration. The conditions were as follows: 50 °C for 10 min, predenaturation at 95 °C for 10 min, then 40 cycles of denaturation at 95 °C for 15 s, and annealing and elongation at 60 °C for 30 s. Relative expression levels were calculated as described previously (Niu *et al.*, 2016). Expression of *At‐β‐TUB4* was used as internal standard. All the primers used for RT‐qPCR are listed in Table S4. Each experiment was repeated three times.

Northern blotting was performed as described previously (Park *et al.*, 2002). In brief, miR472 hybridization was performed using 100 μg total RNA extracted previously, which was separated at 100 V for 6 hr on a denaturing 15% polyacrylamide gel containing 8 M urea. After electroblotting, RNA was transferred to Nytran N nylon blotting membranes (Whatman/GE Healthcare) using a trans‐blot electrophoretic transfer cell (Bio‐Rad). RNA was then bound to the membrane using UV cross‐linking and baking at 80 °C for 1 hr in a UVP HL‐2000 Hybri Linker hybridization oven and crosslinker (Analytik Jena UVP). The α‐^32^P dCTP‐labelled probes were generated using a Prime‐It II Random Primer Labelling Kit (Agilent Technologies). The primers used for probe labelling are listed in Table S4. Hybridization was performed in a hybridization oven at 65 °C overnight and washed according to Hybond's instructions. Radioactive signals were detected using Phosphor Imager screens and developed on a Typhoon Imager (GE Healthcare).

### 4.5 Protein extraction and western blotting

For protein extraction, leaves were collected from seedlings and stored in liquid nitrogen. Total protein was extracted using RIPA lysis buffer (Beyotime). Protein concentration was quantified using a NanoDrop 1000 spectrophotometer (Thermo Scientific). For western blotting, 10 μg total protein was size‐fractionated using 12% sodium dodecyl sulphate‐polyacrylamide gel electrophoresis, then transferred to a Hybond‐P polyvinylidene fluoride (PVDF) membrane (GE Healthcare) using a trans‐blot electrophoretic transfer cell (Bio‐Rad) containing 10% methanol protein transfer buffer. The membrane was probed with a monoclonal anti‐GFP antibody (Roche) and a secondary horseradish peroxidase‐conjugated antibody (anti‐mouse‐HRP) (Beyotime). The horseradish peroxidase was detected using Clarity Western ECL Substrate (Bio‐Rad) on a Bio‐Rad VersaDoc 5000 (Bio‐Rad). Protein expression was quantified using Quantity One 1‐D software (Bio‐Rad). Total proteins on the membrane were stained with 0.1% (wt/vol) Ponceau S, as described previously (Janes, 2015).

### 4.6 Target genes of miR472 prediction and verification

To predict the target genes of miR472, we used psRNATarget (http://plantgrn.noble.org/psRNATarget/), a server for conducting target analysis of plant small RNAs. The guidelines for predicting miR472 targets were set as described by Allen *et al. *(2005). In brief, only one gap with a double penalty was allowed when miR472 was matched with its targets. Inclusion parameters were (a) the nucleotides at positions 10 and 11 of miR472 must match the target completely; and (b) a maximum of three continuous mismatches was allowed if the mismatch region contained at least two G/U pairs. Additionally, the penalty score for the region used for miR472 target selection was less than 3.0 (Allen *et al.*, 2005; Zhang *et al.*, 2011). To verify the actual target genes of miR472, the expression of predicted target genes in the constructed miR472 transgenic lines was analysed using RNA‐Seq data. The expression levels of these genes were detected by RT‐qPCR. An *Agrobacterium‐*mediated tobacco transient expression system was used to simultaneously express miR472 and its targets genes in tobacco. Subsequently, the expression of each target gene and its respective protein product was quantitatively detected using western blotting, GFP fluorescence, and GUS staining.

### 4.7 Construction of RNA‐Seq library and RNA sequencing

In this study, a total of eight samples was obtained from: *A. thaliana* ecotype Col‐0, a line overexpressing miR472, a line bearing a knockdown of miR472, and T‐DNA insertion lines. All samples were obtained using the same growth period, and each seedling was sampled twice. Construction of RNA sequencing libraries was performed using 20 μg total RNA for each sample via a TruSeq RNA Sample Prep Kit v. 2 (Illumina). First, mRNA isolated from the leaves of different lines was purified, dissociated into short fragments, and used to synthesize first‐strand cDNA via a Transcriptor First Strand cDNA Synthesis Kit (Roche). Subsequently, double‐stranded DNA was synthesized using DNA polymerase I and RNase H (Thermo Fisher Scientific), followed by end repair via single‐adenine residue and adapter ligation. The final cDNA library was generated using Phusion High‐Fidelity DNA polymerase (Thermo Scientific). Illumina sequencing was carried out using a HiSeq 2500 platform (Illumina). All sequencing data were saved as FASTQ files and deposited into the NCBI database (BioProject accession number: PRJNA548632; BioSample accession numbers: SAMN12045474, SAMN12045475, SAMN12045476, SAMN12045477, SAMN12045478, SAMN12045479, SAMN12045480, SAMN12045481, SAMN12054380,  and SAMN12054381).

### 4.8 Bioinformatic analysis

#### 4.8.1 Processing of raw reads, de novo assembly, and functional annotation

To obtain sequencing information, raw reads were initially processed and optimized as described previously (Zhang *et al.*, 2015). De novo assembly of the RNA‐Seq library was performed using the Trinity platform (Grabherr *et al.*, 2011). Clean reads were then mapped onto the corresponding contigs, and unigenes with no extension on either end were generated by assembling all the contigs. The assembled unigenes were subjected to similarity search against the NCBI nonredundant protein database via BlastX. Subsequently, Blast annotations were mapped back onto the Uniprot protein database and the GO terms were extracted. To examine putative functions, the assembled unigenes were searched against other databases such as the Swiss‐Prot protein database, GO database, and KEGG pathway database (Wu *et al.*, 2016).

#### 4.8.2 Identification of DEGs and GO enrichment

To quantify the expression of the genes characterized in this study, we mapped RPKM and fragments per kilobase of transcript per million mapped reads. In brief, differential expression analysis was performed using the DESeq2 R package v. 1.24.0, and DEGs were determined via log‐fold expression change (log FC) less than −2 or greater than 2. Venn diagrams were drawn using Venny v. 2.1.0 as described by Oliveros (2007). The KEGG pathway and GO enrichment analyses for all DEGs were conducted using the GOseq R v. 1.36.0 package and KOBAS v. 2.0 plate (Jiang *et al.*, 2019).

### 4.9 Data analysis

All bioassays used in this study were performed at least three times using 48 seedlings per treatment. Analysis of variance was carried out using IBM SPSS Statistics software v. 21.0 (SPSS Inc.). Mean comparison was conducted using a least significant difference (LSD) test ± *SE* or *SD*; *p* < .05 was considered statistically significant.

## CONFLICT OF INTEREST

The authors declare no conflict of interest.

## Data Availability

Sequencing data were deposited in the NCBI BioProject database at https://www.ncbi.nlm.nih.gov/bioproject/, accession number PRJNA548632. Sample information is in the NCBI BioSample database at https://www.ncbi.nlm.nih.gov/biosample/, accession numbers SAMN12045474, SAMN12045475, SAMN12045476, SAMN12045477, SAMN12045478, SAMN12045479, SAMN12045480, SAMN12045481, SAMN12054380, and SAMN12054381.
