# Supplementary figures and images for: Bacillus cereus AR156 triggers induced systemic resistance against Pseudomonas syringae pv. tomato DC3000 by suppressing miR472 and activating CNLs‐mediated basal immunity in Arabidopsis
Source: Mol Plant Pathol. 2020 Mar 30;21(6):854–70. doi: 10.1111/mpp.12935 (PMC7214473; doi:10.1111/mpp.12935)

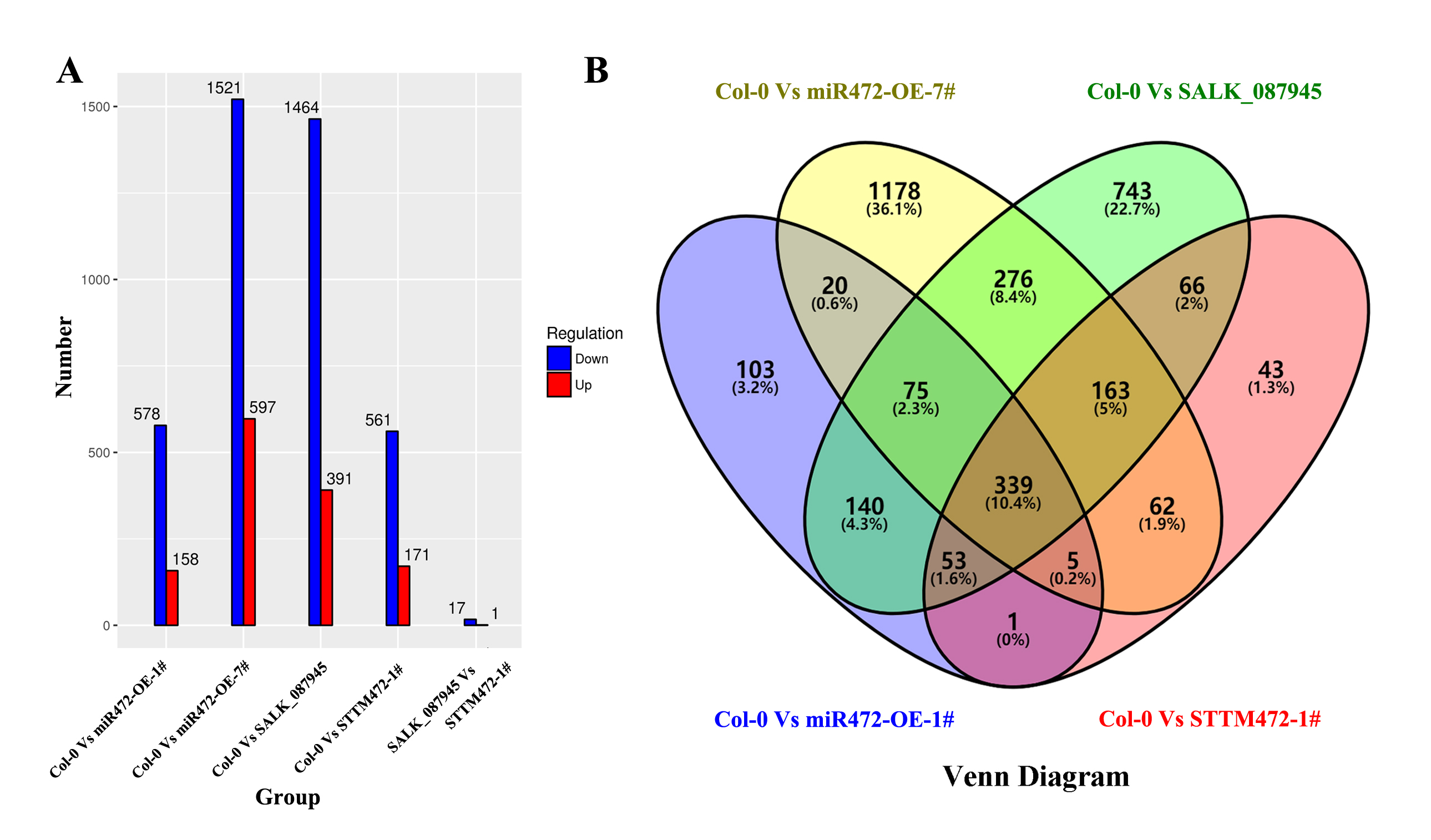

Supplement: Supplementary file 1 [file MPP-21-854-s001.jpg]

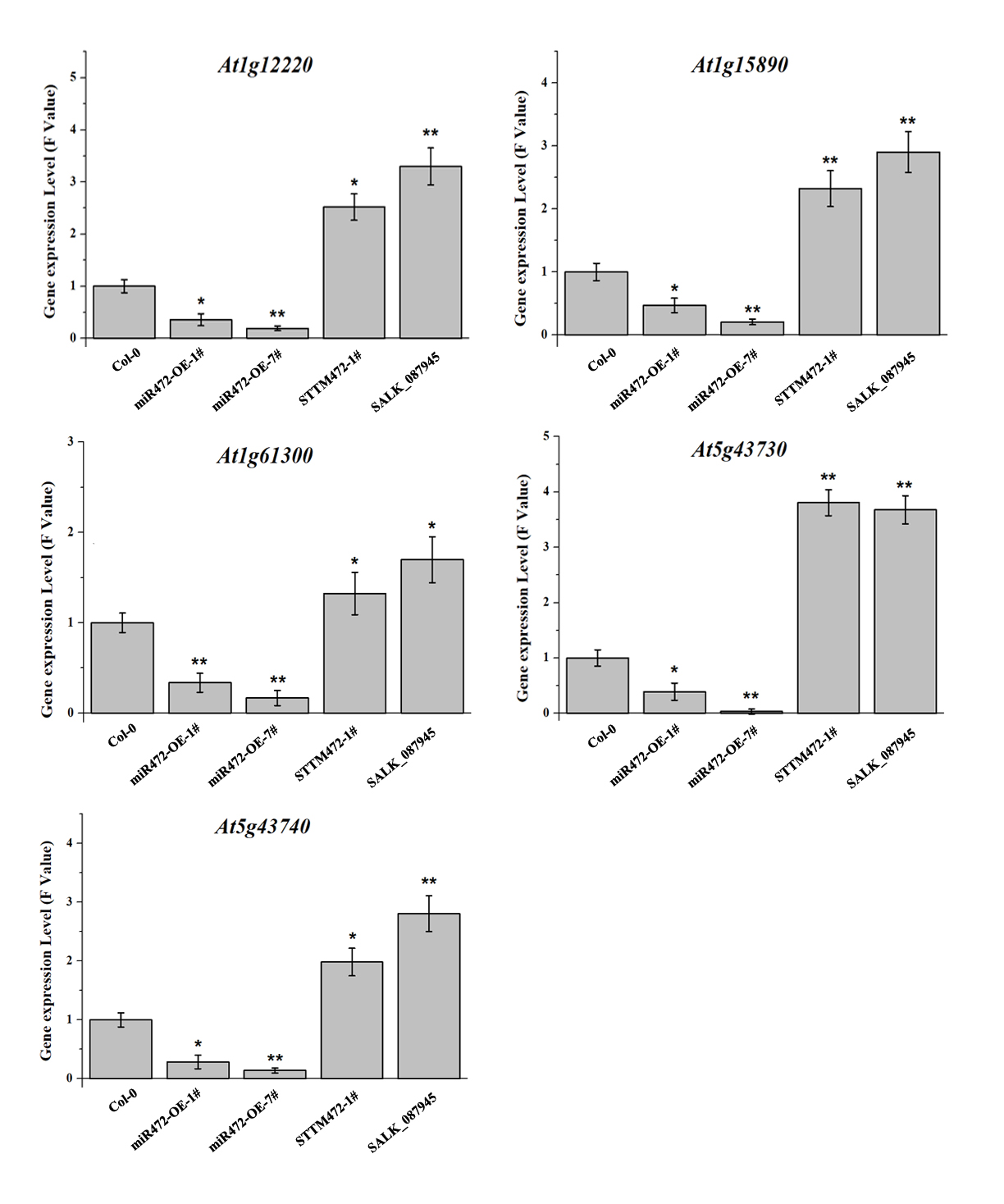

Supplement: Supplementary file 2 [file MPP-21-854-s002.jpg]

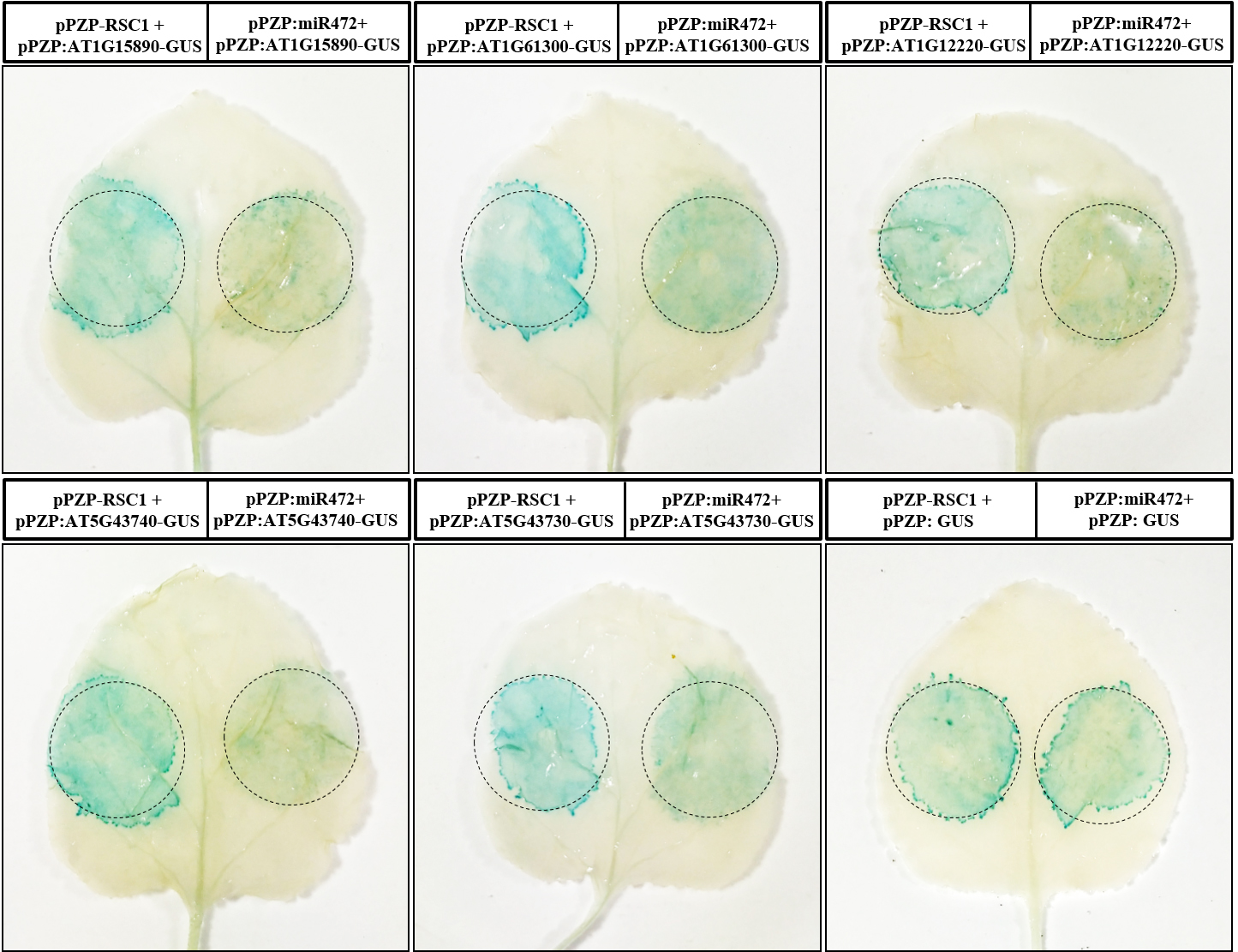

Supplement: Supplementary file 3 [file MPP-21-854-s003.jpg]
